# Ecopiling: a combined phytoremediation and passive biopiling system for remediating hydrocarbon impacted soils at field scale

**DOI:** 10.3389/fpls.2014.00756

**Published:** 2015-01-05

**Authors:** Kieran J. Germaine, John Byrne, Xuemei Liu, Jer Keohane, John Culhane, Richard D. Lally, Samuel Kiwanuka, David Ryan, David N. Dowling

**Affiliations:** ^1^Department of Science and Health, Centre of Research and Enterprise in BioEnvironmental Technologies, The Dargan Centre, Institute of Technology CarlowCarlow, Ireland; ^2^MicroGen Biotech Ltd, Enterprise and Research Incubation Campus, Centre for Research and InnovationCarlow, Ireland; ^3^GES Ltd, Enterprise and Research Incubation Campus, Centre for Research and InnovationCarlow, Ireland

**Keywords:** bioremediation, biopiling, TPH, phytoremediation, field trials

## Abstract

Biopiling is an *ex situ* bioremediation technology that has been extensively used for remediating a wide range of petrochemical contaminants in soils. Biopiling involves the assembling of contaminated soils into piles and stimulating the biodegrading activity of microbial populations by creating near optimum growth conditions. Phytoremediation is another very successful bioremediation technique and involves the use of plants and their associated microbiomes to degrade, sequester or bio-accumulate pollutants from contaminated soil and water. The objective of this study was to investigate the effectiveness of a combined phytoremediation/biopiling system, termed Ecopiling, to remediate hydrocarbon impacted industrial soil. The large scale project was carried out on a sandy loam, petroleum impacted soil [1613 mg total petroleum hydrocarbons (TPHs) kg^-1^ soil]. The contaminated soil was amended with chemical fertilizers, inoculated with TPH degrading bacterial consortia and then used to construct passive biopiles. Finally, a phyto-cap of perennial rye grass (*Lolium perenne)* and white clover (*Trifolium repens*) was sown on the soil surface to complete the Ecopile. Monitoring of important physico-chemical parameters was carried out at regular intervals throughout the trial. Two years after construction the TPH levels in the petroleum impacted Ecopiles were below detectable limits in all but one subsample (152 mg TPH kg^-1^ soil). The Ecopile system is a multi-factorial bioremediation process involving bio-stimulation, bio-augmentation and phytoremediation. One of the key advantages to this system is the reduced costs of the remediation process, as once constructed, there is little additional cost in terms of labor and maintenance (although the longer process time may incur additional monitoring costs). The other major advantage is that many ecological functions are rapidly restored to the site and the process is esthetically pleasing.

## INTRODUCTION

Bioremediation may be defined as the use of biological systems (micro-organisms, plants, or enzymes) to degrade or remove pollutants from contaminated environments. Biopiling, also known as bioheaps, biocells or biomounds, is an *ex situ* bioremediation technology that has been extensively used for remediating a wide range of petrochemical contaminants in soils and sediments (Das and Dash, 2014). Biopiling involves the heaping of contaminated soils/dried sediments into piles and stimulating the biodegrading activity of aerobic microbial populations by creating optimum or near optimum growth conditions within the pile (Jørgensen et al., 2000; Li et al., 2004). This includes the introduction of oxygen through aeration, adjusting pH and moisture levels, and addition of nutrients (nitrogen and phosphorus). As a consequence of these optimum growth conditions, the enhanced microbial activity results in the degradation of the bioavailable organic pollutants (Gomez and Sartaj, 2013). The effectiveness of biopiling has been successfully demonstrated at laboratory and field scale for a number of different types of hydrocarbons (Rojas-Avelizapa et al., 2007; Gomez and Sartaj, 2014; Xu et al., 2014).

Phytoremediation is the use of plants and their associated microbiomes to degrade, sequester or bio-accumulate pollutants from contaminated soil and water. It offers an environmentally friendly, cost effective and carbon neutral approach for the clean-up of toxic pollutants from the environment (Germaine et al., 2012; Khan et al., 2013a; Segura and Ramos, 2013). There have been many studies and reports on the successful use of phytoremediation for the clean-up of sites contaminated with volatile or non-volatile organic pollutants, heavy metals, radioactive compounds, and pesticides. However, the use of plant based technologies does have limitations due to the fact that plants are not ideally suited to the breakdown and metabolism of organic pollutants. As a consequence, phytoremediation can be a very slow process.

To try to address this, as part of our study we describe the combined use of phytoremediation and biopiling in a process termed Ecopiling. Ecopiling is a modification of traditional passive biopiling in that, instead of enclosing the biopile with black plastic, the pile is planted with suitable phytoremediation plants in order to promote rhizoremediation. The Ecopile process involves bio-stimulation of indigenous hydrocarbon degraders, bio-augmentation through inoculation with known hydrocarbon degrading consortia and phytoremediation, through the effect of root growth and penetration throughout the soil and the resulting stimulation of microbial activity in the rhizosphere. Apart from the benefits of having these multi-factorial bioremediation systems for degrading hydrocarbons in impacted soils, Ecopiling has a number of additional benefits including the stabilization of the pile structure by the plant root system and the reduction of leachate production through evapotranspiration. It is much more esthetically pleasing and it returns some of the ecological services back to the site by providing a grassland/meadow habitat.

## MATERIALS AND METHODS

### ECOPILE CONSTRUCTION

**FIGURE 1:**
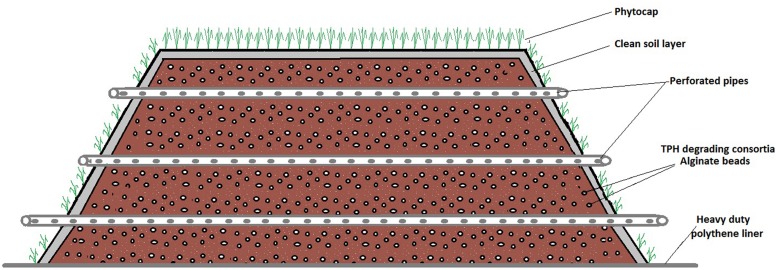
**Systematic of Ecopile design**.

Ecopiling was utilized as a remediation technology on an industrial site in the Republic of Ireland. The site was a former food manufacturing site on which four pockets of mineral oil contaminated subsoil (2–4 m below the surface) were discovered. Over 5000m^3^ of soil was excavated from these contaminated pockets and stockpiled in a disused car park on the site. Nutrients were mixed into the soil in the form of a nitrogen: phosphorus (25:4) fertilizer at a rate of 5 kg per m^3^. The soil was also augmented with a consortium of total petroleum hydrocarbon (TPH) degrading bacteria that had been isolated from contaminated soil on the same site. This consortium was isolated by incubating 10 g of TPH contaminated soil in 500 ml minimal media (Abraham et al., 2002) at 30°C, 100 rpm for 2 weeks and sub-culturing in the same media supplemented with diesel oil every 2 weeks for 3 months. This consortium was immobilized in an alginate bead carrier as described in Power et al. (2011). Typical bacterial numbers in these beads range from 10^8^ to 10^9^ CFU per bead. The alginate beads were applied at a rate of 37 g m^3^ (∼2 x 10^6^ bacteria g^-1^ soil). The Ecopiles were constructed so that they were perpendicular to the prevailing wind. The base layer of soil (0.5 m) was placed over a heavy-duty polythene liner and 50 mm perforated drainage pipe was placed at approximately 1 m centers, laterally across the pile to allow for passive ventilation (**Figure 1**). The Ecopile was then raised in consecutive 0.5 m layers, comprising oil contaminated soil and drainage piping to a height of 2 m. The Ecopiles were constructed trapezoidal in shape with a 2:1 slope from base to top. Finally, each Ecopile was capped with uncontaminated topsoil (∼5 cm deep) and seeded with a 30/30/40 clover, ryegrass, and meadow grass seed mix. Nine Ecopiles were constructed typically 8 m in base width, 4 m in top width, 2 m in height and of various different lengths (20–75 m).

### SAMPLING

Soil samples were taken from Ecopiles 1, 3, 4, 6, and 8, every 3–4 months over a 2 years period. Nine individual samples were taken from each Ecopile and mixed to form one composited sample per Ecopile. These composite samples were analyzed in triplicate for pH, moisture, nitrate, phosphate, total aerobic bacterial counts [total viable count (TVC)], TPH degrader counts and TPH levels. TVC was estimated using standard plate count methods while TPH degraders were estimated using a modified most probable number method (Johnsen et al., 2002) substituting 10 μl of diesel oil for PAHs. Soluble nitrates and phosphates were analyzed using ion chromatography. Total oil/fat contents were determined by Soxhlet extraction on 10 g of soil using 300 ml hexane:acetone (1:1) solvent and refluxing for 24 h. Excess solvent was removed by distillation and the fat/oil content was measured gravimetrically. Soil samples were sent to an independent testing laboratory (Southern Scientific Ltd, Killarney, Ireland) for TPH analysis.

## RESULTS

**FIGURE 2:**
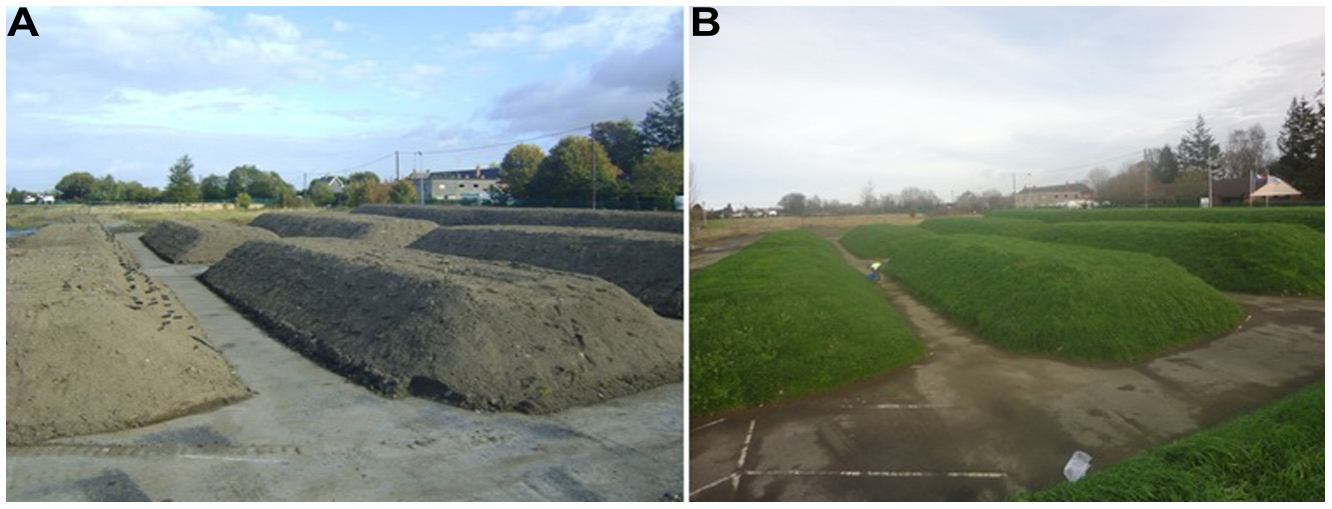
**(A)** Ecopiles after construction in November 2011. **(B)** Ecopiles 1 year after construction in November 2012.

The hydrocarbon impacted soil was generally found to be granular in nature, fine to medium silty sandy (sand 84%, silt 11%, clay 5%) and contained 3.0–9.3% organic matter (mean = 5.72 + 2.17%). Analysis of the soils before Ecopiling by an external commercial laboratory samples showed TPH levels (C_10_–C_40_) of, on average, 1613 mg kg^-1^. In total 4823 m^3^ of contaminated soil was excavated and stockpiled. The contaminated soil was amended with 20,888 kg of chemical fertilizer (24:5 N:P to achieve a 100:10:1 C:N:P ratio) and 190 kg of alginate beads containing the TPH degrading consortium. The soil was used to construct nine Ecopiles (**Figure 2**), the dimensions of which are detailed in **Table 1**.

**Table 1:** Dimensions of the nine biopiles constructed.

Biopile number	Base length (m)	Base width (m)	Height (m)	Estimated volume of soil (m^3^)
1	74.7	12	2.6	1780
2	39	9.2	1.75	488
3	25.3	9.2	2.3	419
4	39	8.7	1.55	418
5	27	9.2	1.65	323
6	41	9.1	1.55	453
7	28.5	9.1	1.65	339
8	25	10	2.4	464
9	14	8.5	1.2	139

*Volumes of Ecopiles were calculated using the following formula: V = L x (b1 + (b2–b1) x h1/h + b1) x h. Where V, volume; b1, base width; b2, top width; h, height*.

**FIGURE 3:**
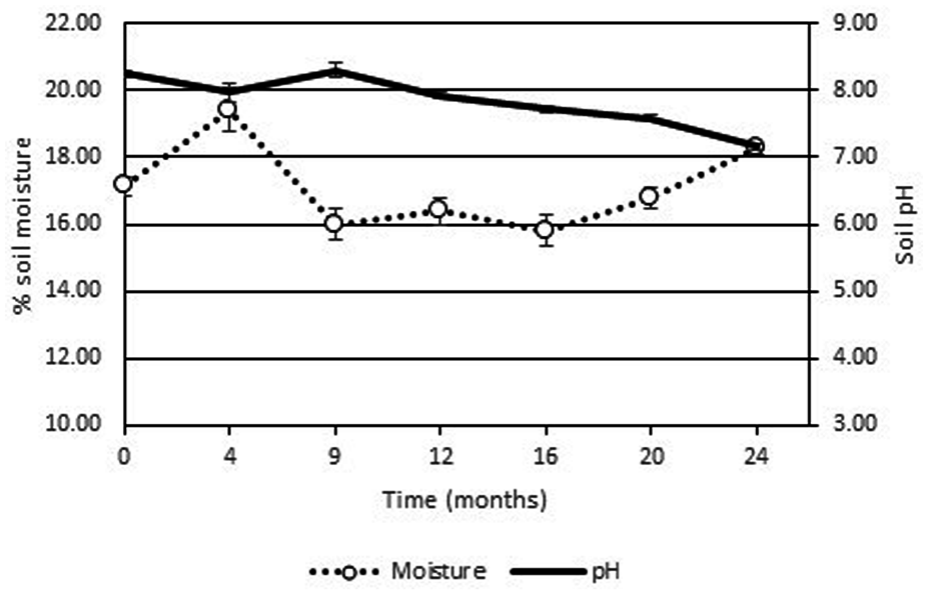
**Average pH and % moisture levels from Ecopile soils over a 2 year period.** Data points represent the mean. Error bars shown are the standard error of the means (*n* = 12).

The high soil pH at the site (pH 7.9–8.7) was due to the presence of lime originating from the food manufacturing process. As the pH levels were just outside of optimum range no pH adjustment of the soil was performed as the addition of the low pH fertilizer would potentially result in lowering the soil pH into the optimum range. The use of ammonium based fertilizers is known to result in a decrease in pH while urea based chemical fertilizers will increase the pH of environmental media (Arnebrant et al., 1990). Indeed the addition of the fertilizer did result in a slow decrease in soil pH over the 2 years period (see **Figure 3**). Soil moisture levels were stable at around 16% over the course of the 2 years period. Although a leachate collection system was constructed, no leachate was generated from the Ecopiles even after heavy rain events. This may have been due to the evapotranspiration of the ryegrass/clover phyto-cap.

**FIGURE 4:**
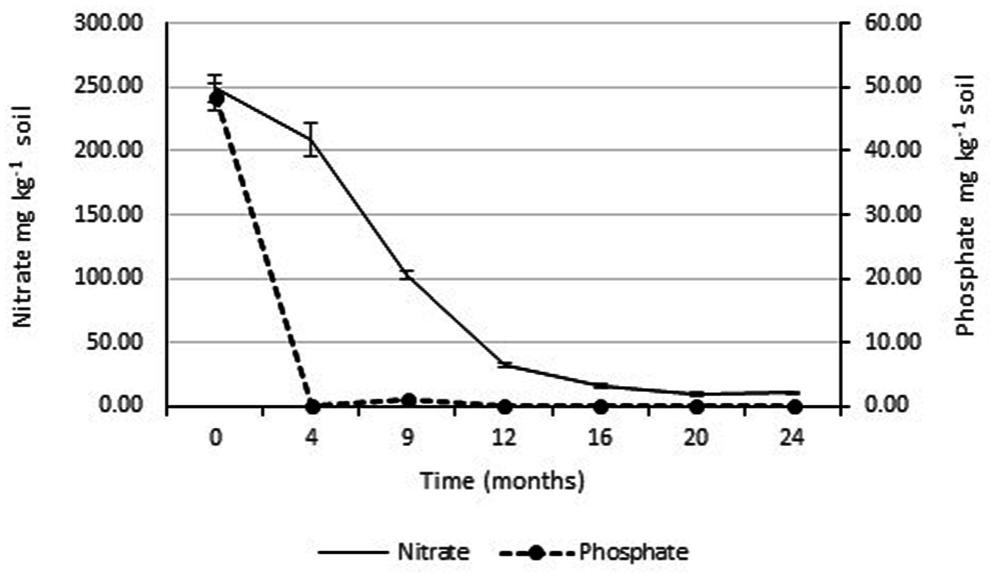
**Average soluble nitrate and phosphate levels within Ecopile soils over a 2 year period.** Data points represent the mean. Error bars shown are the standard error of the means (*n* = 12).

The concentrations of soluble nitrate and phosphate were monitored over the 2 years study period. **Figure 4** shows that there was a rapid drop in soluble phosphate between the time of application and the second monitoring date (3 months later). Soluble phosphate was not detected in any of the soil samples taken at subsequent monitoring dates. Since, there was rapid and continuous growth of the rye-grass/clover phyto-cap, indicating the phosphorus was not limiting, no additional phosphate was applied to the Ecopiles. Soluble nitrate levels also decreased rapidly over the course of the first 12 months dropping from ∼250 to 50 mg kg^-1^ soil. Over the subsequent 12 months, levels of soluble nitrate continued to fall until they reached normal background levels.

**FIGURE 5:**
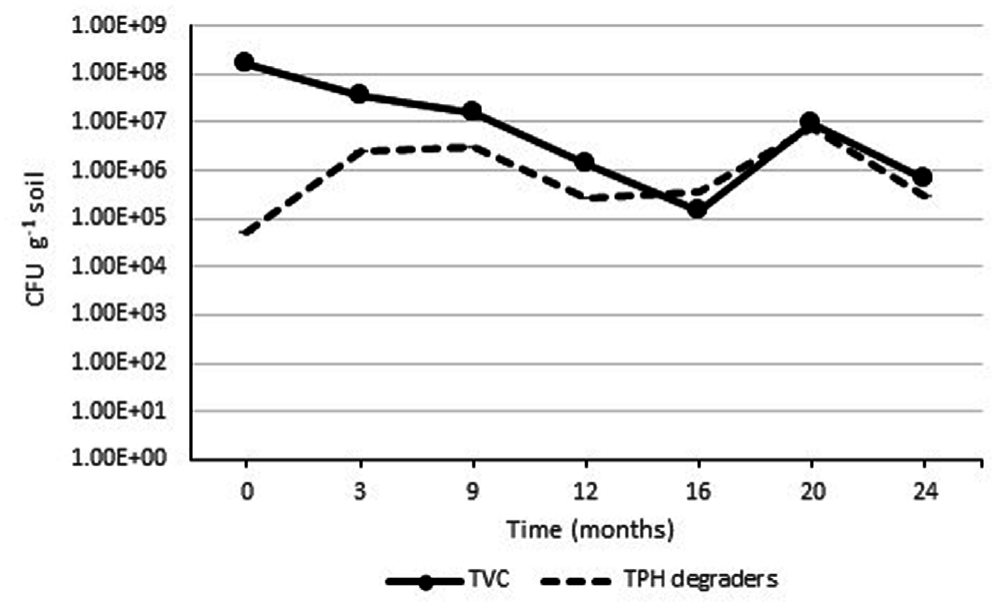
**Average TVC and TPH degrader count within Ecopile soils over a 2 year period.** Data points represent the mean 12 readings. Error bars shown are the standard error of the means (*n* = 12).

Initial analysis of the soil before constructing the Ecopiles showed a relatively low number of aerobic heterotrophic bacteria (1.3 x 10^4^ CFU g^-1^ soil) and a lower number of aerobic oil degrading bacteria (4.2 x 10^2^ CFU g^-1^ soil). This was attributed to the fact that the soil was subsurface material, with a high level of sand. After inoculation with the TPH degrading consortia, nutrient amendment, and construction of the Ecopiles, the TVC increased to an order of 10^8^ CFU g^-1^ soil (**Figure 5**). This increase in bacterial population is likely to have been due to the mixing during Ecopile construction, the stimulating effect of the nutrients added and the inoculation with TPH degrading bacteria. As expected there were seasonal effects on the TVC bacterial population, with decreasing populations during the Autumn/Winter months and increased populations in during Spring/Summer. Similar seasonal trends were observed with the TPH degrader counts where the populations increased in Spring/Summer and decreased in Autumn/Winter. The initial increase in TPH degrader counts may have been partially due to the slow release nature of the alginate bead delivery system that was used to inoculate the contaminated soil during construction.

**FIGURE 6:**
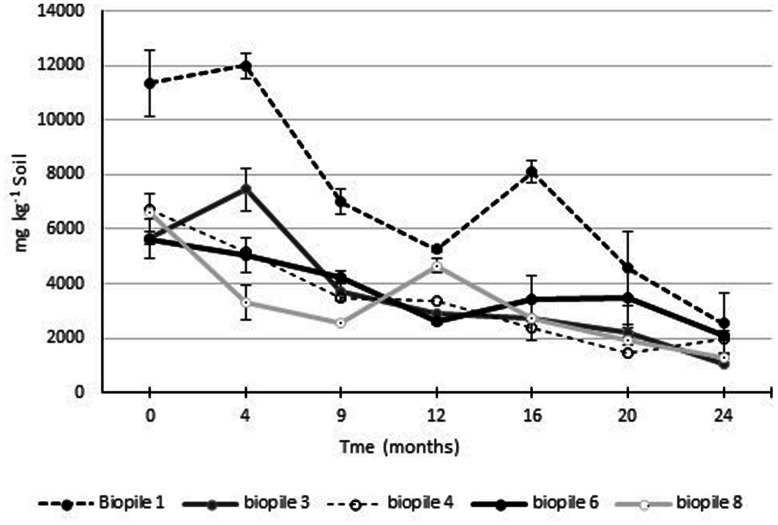
**Levels of Soxhlet extractable fats and oils from Ecopile soils over a 2 year period.** Data points represent the mean of duplicate extractions. Error bars shown are the standard error of the means (*n* = 2).

Gravimetric analysis of Soxhlet extractable fats and oils was used to monitor the process of hydrocarbon degradation in the contaminated soils (**Figure 6**). Ecopile 1 had the highest level of extractable fats/oils at ∼12,000 mg kg^-1^ soil. The soil used to construct this Ecopile originated from the most heavily hydrocarbon impacted location at the site. The remaining nine Ecopiles all resulted in similar quantities of extractable fat/oils ∼5,000–7,000 mg kg^-1^ soil. After 12 months the levels of Soxhlet extractable fat/oils were approximately 50% of those recorded at the start of the study. After a further 12 months Soxhlet extractable fat/oils were in the range of 1050–2500 mg kg^-1^ soil, representing a 62–81% decrease in extractable fats/oils. Analysis of the soils 24 months after the Ecopiling process had started, by an external laboratory showed that TPH concentrations (both aliphatic and aromatic C_12_–C_40_) in eight out of the nine Ecopiles were below detectable limits. The only exception was that one of the three samples taken from Ecopile 1 contained TPH levels of 152 mg kg^-1^ soil in the C_16_–C_35_ range.

**FIGURE 7:**
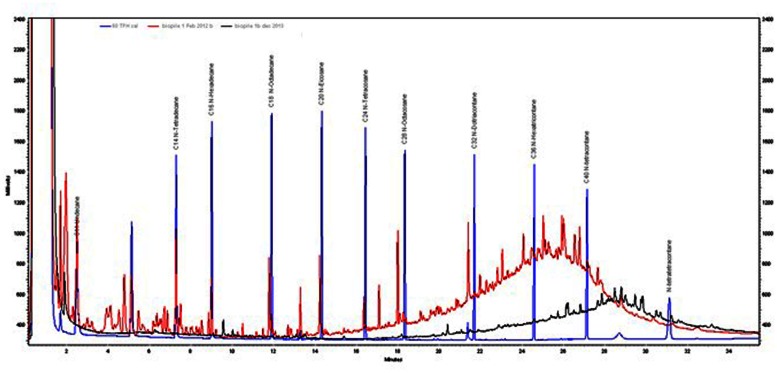
**GC profiles of Soxhlet extractions from biopile 1 soil in February 2012 (red trace) and November 2013 (black trace)**. TPH standard (blue trace) is included for comparison.

GC-FID profiles of the Soxhlet extracts showed that almost 100% of low boiling point hydrocarbons (<C20) had been removed after 24 months (**Figure 7**). These fractions tend to be the more water soluble and toxic components of mineral oils. Therefore after 12 months the toxicity of the soil is likely to have been significantly reduced. The heavier fractions had been reduced by 67–80% after 24 months.

## DISCUSSION

Biopiling has been used successfully to treat crude, diesel and lubrication oil contaminated soil. Mao et al. (2009) used biopiling with active aeration and nutrient amendment to remediate soil which was grossly contaminated with mineral oil (10,000–20,000 mg kg^-1^ D.M). After 18 weeks TPH levels were shown to be reduced by 80%. In their study they constructed relatively small biopiles and the aeration process used involved mixing the biopile soil every 2 weeks. This method may not be logistically and economically feasible for very large volumes of soil. In contrast, Coulon et al. (2010) found that windrowing was far more effective than biopiling at removing TPH from soil contaminated with bunker fuel. They reported that there was rapid removal of TPH from the windrowed treatment, with almost 50% degradation after just 5 weeks and after 20 weeks TPH levels were just 2–4% of the initial levels. Whereas in their biopiles, TPH degradation was much more gradual, after 20 weeks TPH levels had dropped by 50% and at the end of their 30 weeks trial TPH levels were down to 22% of initial levels. In a study carried out by Wang et al. (2012) 50% degradation of TPH was observed after 220 days in biopiles used to treat oily sludge. With the Ecopile system, 50% degradation was not achieved until 52 weeks into the remediation project. Although the Ecopile system does result in slower contaminant degradation rates, it is significantly less costly and labor intensive than active aeration and windrowing systems.

The main mechanism of contaminant removal in biopiles is through the stimulation of the metabolic activities of hydrocarbon degrading microbes. This activity is stimulated by the addition of nutrients and through enhancing oxygen diffusion through the soil. Soil microbial activity can also be enhanced in biopile soil by means of the direct addition of hydrocarbon degrading inocula (Lebkowska et al., 2011; Chemlal et al., 2012). However, recent studies indicate controversy over the benefits of nutrient amendments in hydrocarbon remediation. Sarkar et al. (2005) found that there was no statistical difference in TPH degradation rates among nutrient amended and unamended soil. However, they did show that microbial activity increased in contaminated soils ammended with biosolids and inorganic fertilizers. Based on their results they recommended that biosolids be used as the fertilizers in bioremediation projects as inorganic fertilizers can reduce microbial activity due to toxicity from high levels of ammonia. Inhibition of oil degradation caused by high N additions was also reported by Akbari and Ghoshal (2014). Coulon et al. (2010) also observed very little difference in TPH degrader counts between their control soil (with no amendments), soil with nutrients added and soil that had nutrients added and had been inoculated with TPH degraders. Likewise, Bento et al. (2005) observed very little effect of bio-stimulation and bio-augmentation on the TPH degrader counts and TVC levels in oil contaminated soil, while Wang et al. (2012) observed a negative effect on TPH numbers after the addition of nutrients to the soil. On the other hand, Lin et al. (2010) reported that the total heterotrophic counts increased in oil contaminated soil that had been amended with nutrients. They observed an initial rapid increase in populations and then a slow decline to approximately 10^5^ CFU/g soil at the end of their trial.

Inoculation of the soil in the current remediation project was deemed necessary as there was a low microbial population to begin with. The guidelines of the US EPA suggest that bioremediation is feasible when there are about 10^3^ CFU/g soil (Lin et al., 2010). Again the usefulness of soil inoculation with hydrocarbon degrading microbes is also a focus of debate among researchers. Jørgensen et al. (2000) tested two commercially available inocula when biopiling lubrication oil contaminated soil. They found no statistical difference in oil degradation rates between inoculated and uninoculated biopiles. Whereas Kim et al. (2005), found that inoculation of soil with oil degraders did enhance the metabolic activity in the test soil. Immobilization of the inocula in natural or synthetic polymers is known to reduce competition from indigenous soil microbes, reduce predation and allows the inocula time to adapt to soil conditions such as pH (Cunningham et al., 2004). Cunningham et al. (2004) found that immobilization of the oil degrading consortia in Polyvinyl gels lead to enhanced oil degradation rates in soil compared to the application of free living cells. They also demonstrated that the inoculation of on an enriched culture of autochthonous micro-organisms was more effective than bio-stimulation with inorganic nutrients and aeration with bulking agents. The usefulness of encapsulation was also demonstrated by Power et al. (2011) when introducing PCB-degrading and biosensing bacteria into contaminated soils. There are also many reports describing the benefits of using plants to stimulate microbial activity in contaminated soils. Khan et al. (2013b) showed that planting rye grass (*Lolium multiflorum*) in diesel contaminated soil increased the rate of TPH degradation by 25% compared to unplanted soil. They also showed that soil planted with ryegrass and inoculated with various diesel degrading bacteria resulted in TPH degradation rates of 67–84% greater than that seen in control soils.

## CONCLUSION

To our knowledge this is the first report of combining phyto-remediation with a passive biopiling process, which we termed Ecopiling. Ecopiling is a multi-factorial bio-remediation process involving bio-stimulation, bio-augmentation and phyto-remediation. The process is most suitable for remediation projects with large volumes of contaminated soil, sufficient space available to construct the Ecopiles, and most importantly, have medium to long term time- scales (2–4 years). Many former abandoned industrial and crude oil contaminated sites fit in to this category. One of the key advantages to the process is the reduced cost of the remediation process. Once the Ecopiles are constructed, there is little additional cost in terms of labor and maintenance, although the longer process time may incur additional monitoring costs. The growth of the plants on the Ecopiles rapidly returns some of the ecological functions to the site, while the bioremediation process is ongoing.

## AUTHOR CONTRIBUTIONS

Kieran J. Germaine: was involved in the trial concept design, project management, and data acquisition and responsible for preparation of the manuscript; John Byrne was involved in the trial design, project management, data acquisition, and reviewing the manuscript; John Culhane, Richard D. Lally, Samuel Kiwanuka were involved in data acquisition and reviewing the manuscript; Xuemei Liu, Jer Keohane, David Ryan, and David N. Dowling were involved in pre-trial analysis, initial testing, Ecopile design and reviewing the manuscript.

## Conflict of Interest Statement

Envirocore and GES Ltd were renumerated by the owners of the industrial site for the services of designing and monitoring the Ecopiles described in this manuscript. The project had oversight from the Irish Environmental Protection Agency as part of the industrial sites remedial action plan under its waste management license.
